# A Laboratory-Friendly CTC Identification: Comparable Double-Immunocytochemistry with Triple-Immunofluorescence

**DOI:** 10.3390/cancers14122871

**Published:** 2022-06-10

**Authors:** Raed Sulaiman, Pradip De, Jennifer C. Aske, Xiaoqian Lin, Adam Dale, Ethan Vaselaar, Nischal Koirala, Cheryl Ageton, Kris Gaster, Joshua Plorde, Benjamin Solomon, Bradley Thaemert, Paul Meyer, Luis Rojas Espaillat, David Starks, Nandini Dey

**Affiliations:** 1 Physicians Laboratory, Department of Pathology, Avera McKennan Hospital & University Health Center, Sioux Falls, SD 57105, USA; raed.sulaiman@plpath.org; 2 Translational Oncology Laboratory, Avera Research Institute, Sioux Falls, SD 57105, USA; pradip.de@avera.org (P.D.); jennifer.aske@avera.org (J.C.A.); xiaoqian.lin@avera.org (X.L.); adam.dale@avera.org (A.D.); ethan.vaselaar@avera.org (E.V.); nischal.koirala@avera.org (N.K.); 3 Department of Internal Medicine, University of South Dakota SSOM, USD, Sioux Falls, SD 57105, USA; 4 Department of Research Oncology, Clinical Research, Sioux Falls, SD 57105, USA; cheryl.ageton@avera.org; 5 Avera Cancer Institute, Avera McKennan Hospital, Sioux Falls, SD 57105, USA; kris.gaster@avera.org; 6 Diagnostic Radiology, Interventional Radiology, and Radiology, Avera Medical Group Radiology, Sioux Falls, SD 57105, USA; joshua.plorde@avera.org; 7 Hematology and Oncology, Avera Medical Group Oncology & Hematology, Sioux Falls, SD 57105, USA; benjamin.solomon@avera.org; 8 Bariatrics, Surgery, and General Surgery, Surgical Institute of South Dakota, Sioux Falls, SD 57105, USA; bradley.thaemert@avera.org; 9 Cardiovascular/Thoracic Surgery, Surgery North Central Heart, A Division of Avera Heart Hospital, Sioux Falls, SD 57105, USA; pmeyer@ncheart.com; 10 Department of Gynecologic Oncology, Avera Cancer Institute, Sioux Falls, SD 57105, USA; luis.rojasespaillat@avera.org (L.R.E.); david.starks@avera.org (D.S.)

**Keywords:** CTC, immunocytochemistry, *parallel double-detection*, laboratory-friendly

## Abstract

**Simple Summary:**

Tumor cells that circulate in the peripheral blood of patients with solid tumors are called circulating tumor cells. Since the source of circulating tumor cells are from primary cancer sites, metastatic sites, and/or a disseminated tumor cell pool, these cells have clinical significance. The circulating tumor cells offer a rare glimpse of the evolution of the tumor and its response/resistance to treatment in a real-time non-invasive manner. Although the clinical relevance of circulating tumor cells is undeniable, the routine use of these cells remains limited due to the elusive nature of the cells, which demands highly sophisticated and costly instrumentation. We presented a specific and sensitive laboratory-friendly *parallel double-detection format* method for the simultaneous isolation and identification of circulating tumor cells from peripheral blood of 91 consented and enrolled patients with tumors of the lung, endometrium, ovary, esophagus, prostate, and liver. Our user-friendly cost-effective circulating tumor cells detection technique has the potency to facilitate the routine use of circulating tumor cells detection even in community-based cancer centers for prognosis, before and after surgery, which will provide a unique opportunity to move cancer diagnostics forward.

**Abstract:**

The source of circulating tumor cells (CTC) in the peripheral blood of patients with solid tumors are from primary cancer, metastatic sites, and a disseminated tumor cell pool. As 90% of cancer-related deaths are caused by metastatic progression and/or resistance-associated treatment failure, the above fact justifies the undeniable predictive and prognostic value of identifying CTC in the bloodstream at stages of the disease progression and resistance to treatment. Yet enumeration of CTC remains far from a standard routine procedure either for post-surgery follow-ups or ongoing adjuvant therapy. The most compelling explanation for this paradox is the absence of a convenient, laboratory-friendly, and cost-effective method to determine CTC. We presented a specific and sensitive laboratory-friendly *parallel double-detection format* method for the simultaneous isolation and identification of CTC from peripheral blood of 91 consented and enrolled patients with various malignant solid tumors of the lung, endometrium, ovary, esophagus, prostate, and liver. Using a pressure-guided method, we used the size-based isolation to capture CTC on a commercially available microfilter. CTC identification was carried out by two expression marker-based independent staining methods, double-immunocytochemistry parallel to standard triple-immunofluorescence. The choice of markers included specific markers for epithelial cells, EpCAM and CK8,18,19, and exclusion markers for WBC, CD45. We tested the method’s specificity based on the validation of the staining method, which included positive and negative spiked samples, blood from the healthy age-matched donor, healthy age-matched leucopaks, and blood from metastatic patients. Our user-friendly cost-effective CTC detection technique may facilitate the regular use of CTC detection even in community-based cancer centers for prognosis, before and after surgery.

## 1. Introduction

Circulating tumor cells (CTCs) are rare and heterogeneous cellular components circulating in the peripheral blood of patients with solid tumors [1] and are considered one of the fundamental elements of the blood-based biopsy. As the source of CTCs in the bloodstream has been known to be from primary cancer sites, secondary metastatic sites, and/or a disseminated tumor cell pool, the predictive [2] and prognostic [2,3] values of CTC have been established in most solid tumors including prostate [4], hepatocellular [5], breast [6,7,8], colorectal [9,10] melanoma [11], head and neck [12], bladder [13], testicular [14], and gastric cancers [15] in both localized and metastatic clinical settings [1]. The prognostic and therapeutic implications of CTC phenotype detection based on epithelial–mesenchymal transition markers in the first-line chemotherapy of HER2-negative metastatic breast cancers indicated the role of CTCs in the management of the disease [16]. CTC enumeration has also proven its potential to improve the management of cancers in several other ways. The value of real-time longitudinal CTC fluctuations can provide the opportunity for (1) treatment intensification in patients with a poor prognosis or (2) de-escalation in patients with a good prognosis. CTC as an endpoint has the potential to evaluate the efficacy of treatment alongside the molecular characteristics of CTCs, which provides their theranostic value [3]. The utility of CTCs as a multifunctional biomarker focusing on their potential as pharmacodynamic endpoints either directly via the molecular characterization of specific markers or indirectly through CTC enumeration has been reported [17].

In spite of the well-recognized clinical validity and utility [18] of enumerating CTC in nonmetastatic and metastatic cancers [3], the determination of CTC as a routine strategic procedure is yet to be incorporated into standard clinical practice for the management of the disease [17]. Studies involving the treatment based on (1) CTC count, (2) CTC variations, and/or (3) the molecular characteristics of CTCs were sometimes inconclusive or are still ongoing [3]. One of the reasons CTC determination does not serve as a routine standard liquid biopsy in patients with solid tumors has been identified as the lack of much-needed improvement in the method to test CTC [19]. We need user-friendly, cost-effective, yet reproducible methods to determine CTC routinely for diagnostic (especially with germline mutation or predisposition), predictive, and prognostic purposes across different cancer centers, including community-based hospitals.

CTCs are a promising yet challenging tumor biomarker to detect. The road-block is a methodological issue, as a clinically dependable enumeration of CTC is still limited to primarily established resource-rich, comprehensive centers employing sophisticated instrumentation. Here we presented a low-cost, specific, sensitive, and fail-safe laboratory-friendly method for simultaneous isolation and identification of CTC from 91 consented and enrolled patients with various solid tumors, including lung, endometrial, ovarian, esophageal, prostate, and liver cancers.

## 2. Methods

### 2.1. Cell Lines and Reagents

Cell lines from endometrial, ovarian, breast, and lung cancers (AN3CA, Cat # HTB-111; RL-95-2, cat # CRL-1671; OVCAR3, cat # HTB-161; MCF7, cat # HTB-22; HCC1975, cat # CRL-5908 and NCI-H441 cat # CRM-HTB-174), human uterine fibroblasts (HUF; Primary Uterine Fibroblasts, Cat # PCS-460-010), and HUVEC cells were procured from ATCC (cat # PCS-100-013) and were cultured according to the standard cell culture procedures as per ATCC recommendations. Leucopak, PBMC (peripheral blood mononuclear cells) were procured from Lonza (Lonza Group Ltd., Basel, Switzerland). The CellSieve enumeration kit with either DAPI/CK-FITC/EpCAM-PE/CD45-Cy5 or DAPI/CK-FITC/CD31-PE/CD45-Cy5 was procured from Creatv Microtech.

### 2.2. Patients & Blood Collection

All experimental protocols were approved by the institutional and/or licensing committee/s. The informed consent(s) was obtained from all subjects and/or their legal guardian(s). Informed (IRB approved: Protocol Number Study: 2017.053-100399_ExVivo001) consents for obtaining the peripheral blood were obtained from 91 enrolled patients with various solid tumors, including lung, endometrial, ovarian, esophageal, prostate, and liver cancers. All methods were carried out in accordance with relevant guidelines and regulations. Blood samples were collected in commercially available CellSave collection tubes (Menarini Silicon Biosystems, Bologna, Italy) [20]. We included samples from patients with solid tumors at any stage/grade of the disease undergoing surgery/biopsy with or without pre-treatment/history of any previous carcinoma. We did not include any bone-marrow transplant patients or patients with liquid tumors.

### 2.3. Isolation and Enrichment of CTCs

The isolation and size-based enrichment of CTCs from blood was achieved by (CellSieve^TM^; Creatv Microtech, Potomac, MD, USA) using precision, high-porosity lithographic microfilters (high capture efficiency precision CellSieve^TM^ microfilters of biocompatible polymer with dense, uniform pores) [21,22,23]. Size-based filtration was carried out to eliminate red blood cells differentially and most white blood cells from whole blood, retaining larger cells on the surface of the filter [24] using a syringe pump (KD Scientific Legato 110 CMT; Analytical West, Inc., Lebanon, PA, USA) assembled with filter holder assembly (Creatv Microtech; Potomac, MD, USA).

### 2.4. Identification of CTCs by Double-Immunocytochemistry Assay

We seamlessly coupled the isolation and enumeration of CTC by double-immunocytochemistry staining. The entire procedure of the CK8,18^+^/CD45^−^ (staining for CK8,18 positivity and CD45 negativity) double immunocytochemistry (ICC×2), from permeabilization to counterstaining, was carried out on a microfilter installed in the syringe pump. The isolated cells on the microfilter were permeabilized by a dual endogenous enzyme blocking buffer with 0.3% hydrogen peroxide-containing sodium azide and levamisole (DAKO; EnVision^®^+ Dual Link System-HRP (DAB+). Code K4065). Following washing with TBST, pH 7.1, the microfilter was incubated for 1 h at room temperature in 600–700 microliters of a mixture of 1:6000 diluted mouse mAb cytokeratin 8 and 18 (B22.1 & B23.1) (Cell Marque^TM^ Tissue Diagnostic, Millipore-Sigma; Cat. Number: 818M-90) and 1:800 diluted rabbit mAb CD45 (Cell Signaling Technology; D9M8I XP; Catalog # 13917) primary antibodies. Following washing (×3) with TBST, pH 7.1, the microfilter was incubated for 35–40 min at room temperature in 200–300 microliters of a 1:1 mixture of secondary rabbit-Ab-AP-Polymer (Abcam DoubleStain IHC Kit: M&R on human tissue (DAB and AP/Red) Cat. # ab210059) and secondary mouse-Ab-HRP-Polymer (Abcam DoubleStain IHC Kit: M&R on human tissue (DAB and AP/Red) Cat. # ab210059) under light-protected conditions. We used DAKO 10× wash buffer (pH 7.6) (DAKO Wash Buffer 10×; Code S300685-2C) supplied as a 1 L concentrated Tris-buffered saline solution (10×) containing Tween 20, pH 7.6 (±0.1) for washing. Following washing (×3) with DAKO wash buffer, the color was developed using DAB (3,3’-diaminobenzidine chromogen) reagents, DAB substrate buffer pH 7.5, and DAB+ chromogen (DAKO; EnVision^®^+ Dual Link System-HRP (DAB+) Code K4065). The chromogenic reaction was stopped by washing (×1) in DD water. DAB color was monitored under a microscope following washes (×3) in DAKO washing buffer. The chromogenic reaction of the alkaline-phosphatase was prepared using permanent Red-Substrate, permanent Red-Activator, and permanent Red-Chromogen (Abcam; Ab210059). Then, 200–300 microliters of the reconstituted final solution were used for incubation (×2) for 20 min. Following washing (×3) with DD water, the cells were counterstained (×2) with filtered DAKO hematoxylin (DAKO; Code S3302) for 10–15 min. Hematoxylin color was developed by incubating the microfilter for 3 min each time and washing using 30 mL of DD water. The air-dried membrane was mounted in a resin-based permanent non-aqueous mounting media (Richard Allan Scientific Mounting Media (Thermo Fisher Scientific: Catalog # 4111TS-TS). For ICC×2, pictures were taken at 40× objective of Olympus BX43 Microscope using cellSens 1.18 LIFE SCIENCE IMAGING SOFTWARE (OLYMPUS CORPORATION).

### 2.5. Parallel Identification of CTCs by Triple-Immunofluorescence Assay to Validate ICC×2

**Figure 1:**
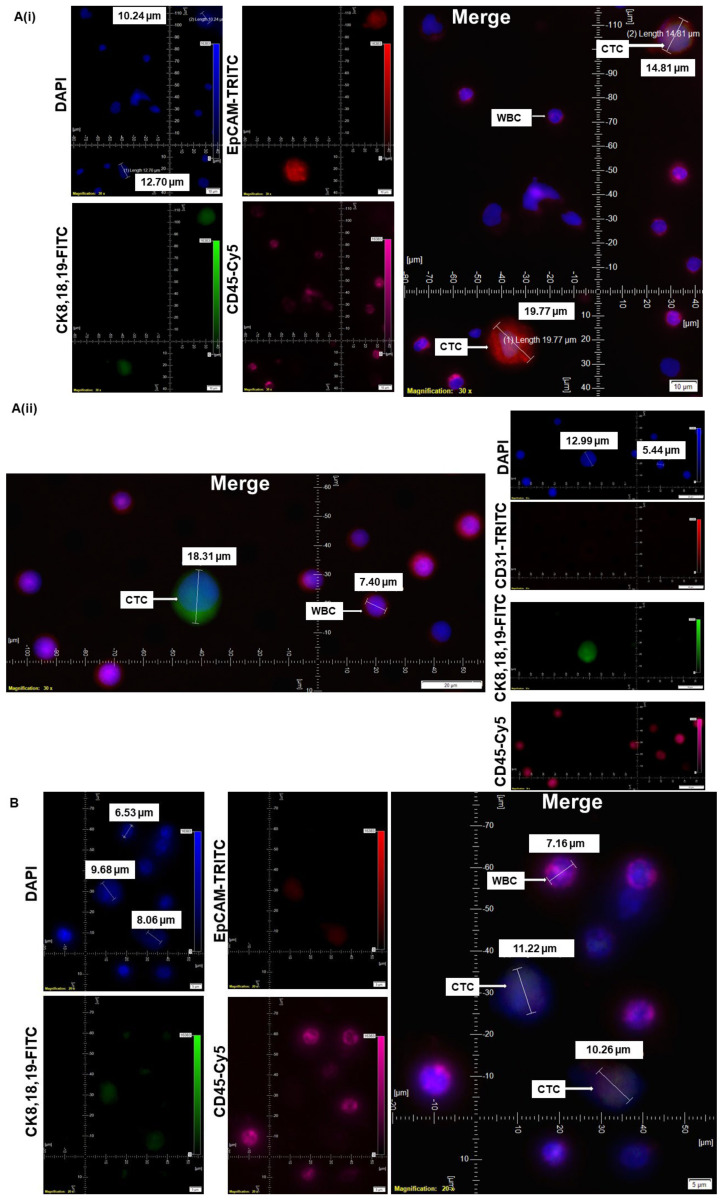

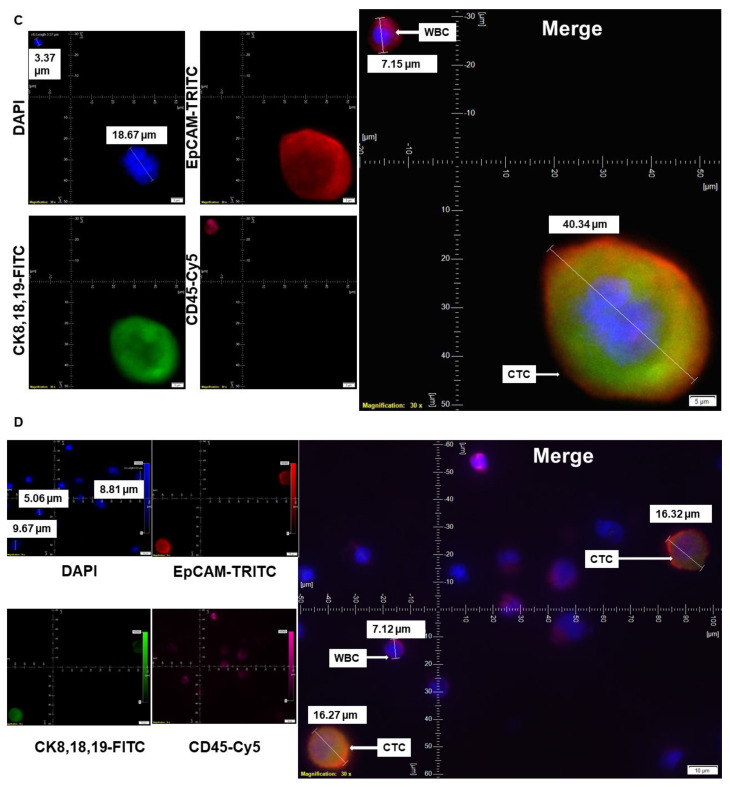
Standardization and validation of CTC by IF×3 using breast, ovarian, and lung cancer cell lines: Patients’ blood samples spiked with titrating number (1000 cells, 750 cells, 375 cells, 250 cells/100 cells) of cell lines of different solid tumors using. Pictures were taken at 60× oil objective of an Olympus IX71 Microscope with DAPI/FITC/TRITC/CY5 filter sets. (**A**): MCF7 cells (750 cells/375 cells per 7.5 mL of patient’s blood) were used for spiking blood samples, and cells were captured on a microfilter and stained with a CellSieve enumeration kit (Creatv Microtech) with either DAPI/CK-FITC/EpCAM-PE/CD45-Cy5 (**Ai**) or DAPI/CK-FITC/CD31 PE/CD45-Cy5 (**Aii**). (**B**): OVCAR3 cells (100 cells per 7.5 mL of patient’s blood) were used for spiking blood samples, and cells were captured on a microfilter and stained with cell sieve enumeration kit (Creatv MicroTech) with DAPI/CK-FITC/EpCAM-PE/CD45-Cy5. (**C**): HCC1975 cells (1000 cells per 7.5 mL of patient’s blood) were used for spiking blood samples, and cells were captured on a microfilter and stained with cell sieve enumeration kit (Creatv Microtech) with DAPI/CK-FITC/EpCAM-PE/CD45-Cy5. (**D**): NCI-H441 cells (250 cells per 7.5 mL of patient’s blood) were used for spiking blood samples, and cells were captured on a microfilter and stained with cell sieve enumeration kit (Creatv Microtech) with DAPI/CK-FITC/EpCAM-PE/CD45-Cy5. The magnification, scale bar, and digital reticle are represented for each photomicrograph. Fluorescence images from DAPI, FITC, TRITC, and Cy5 channels were separated as pictures with a color bar. The fluorescence-photomicrographs presented the diameters (μm) of CTC and a representative WBC and their respective DAPI stained nucleus.

**Figure 2:**
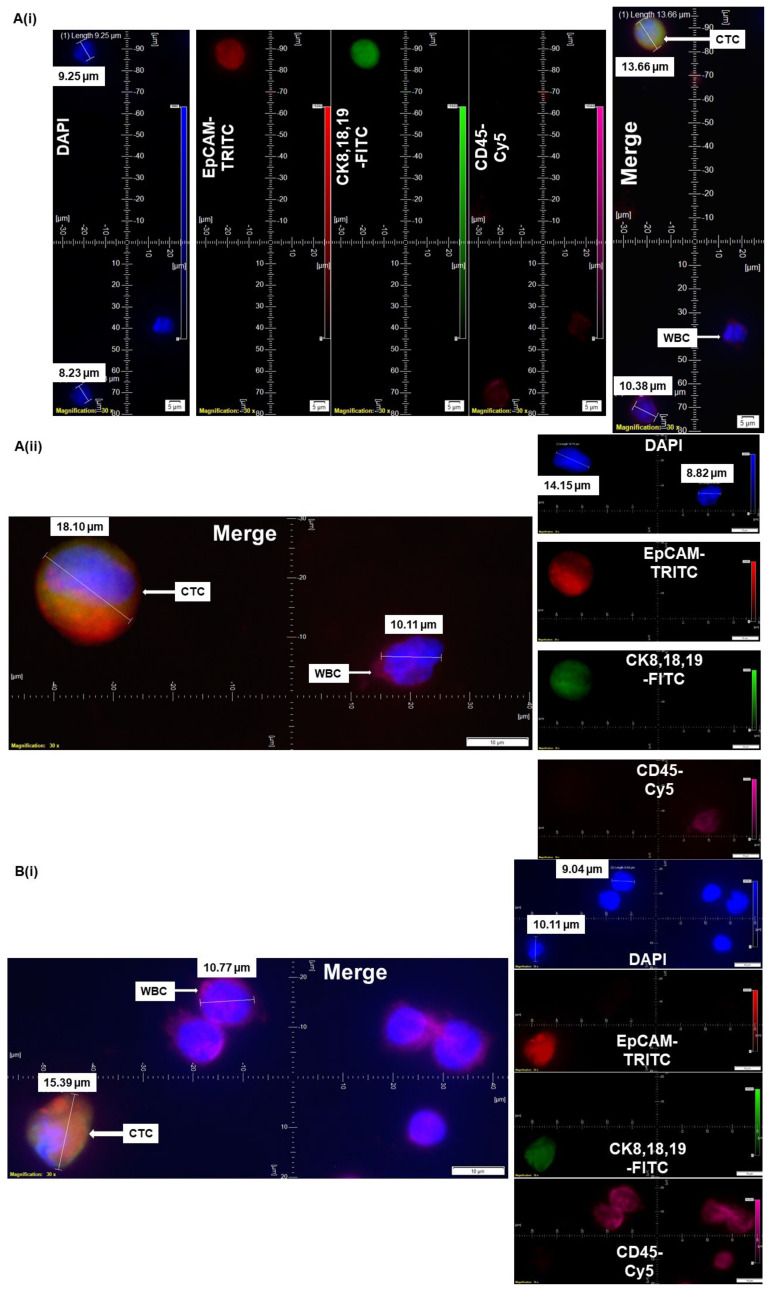

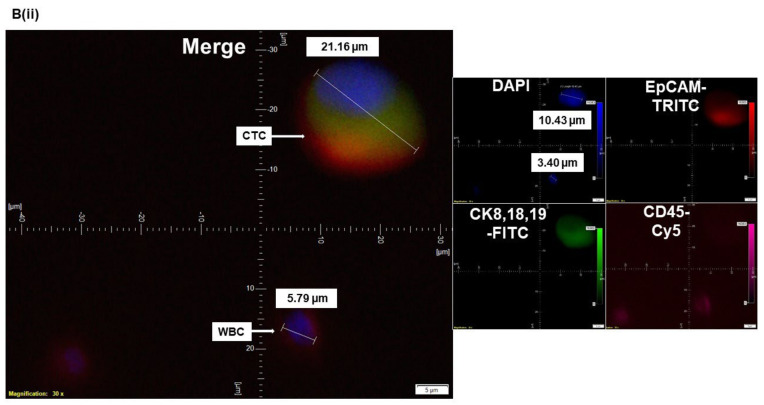
Validation spectrum of CTC by IF×3 using blood from patients with different clinical statuses and samples of origin: CTC from blood samples from patients with (**A**) clinical status, nonmetastatic (**Ai**) and metastatic (**Aii**) in endometrial cancers, and (**B**) samples of origin, during a biopsy from a patient with metastatic liver cancer (**Bi**) and during surgical resection of the tumor in lung cancers (**Bii**) are presented. The magnification, scale bar, and digital reticle are presented for each photomicrograph. Fluorescence images from DAPI, FITC, TRITC, and Cy5 channels were separated as pictures with a color bar. The fluorescence-photomicrographs presented the diameters (μm) of CTC and a representative WBC and their respective DAPI stained nucleus.

**Figure 3:**
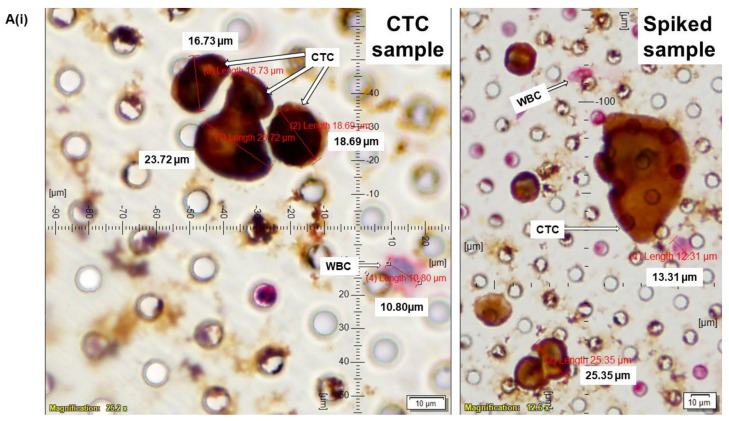

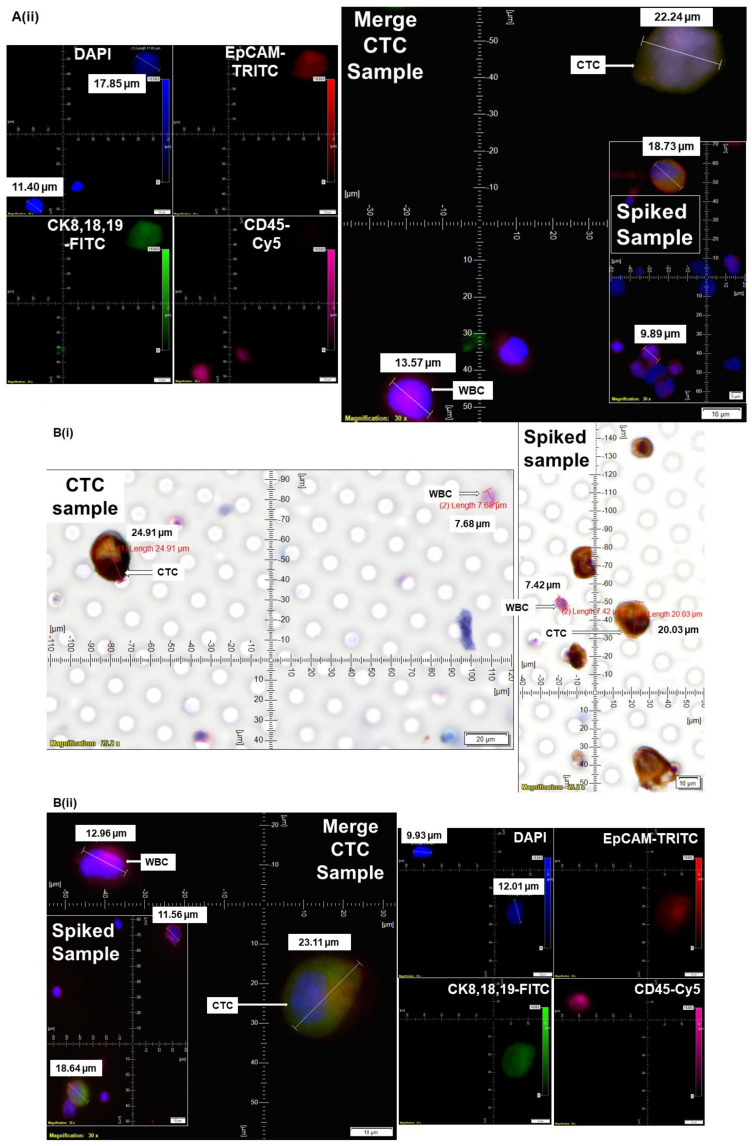
Standardization and validation of CTC by ICC×2 in reference to spiked IF×3 in endometrial and ovarian cancers: CTCs were captured from blood samples from patients with endometrial (**A**) and ovarian (**B**) tumors and enumerated using ICC×2 (**Ai**,**Bi**) in reference to IF×3 (**Aii**,**Bii**). Blood samples were spiked (Spiked samples) with titrating numbers (250 cells/100 cells) of NCI-H441 cells separately for both ICC×2 and IF×3. For IF×3, pictures were taken at 60× oil objective of an Olympus IX71 Microscope with DAPI/FITC/TRITC/CY5 filter sets. For ICC×2, pictures were taken at 40× objective of an Olympus BX43 Microscope. The magnification, scale bar, and digital reticle are represented for each photomicrograph. Fluorescence images from DAPI, FITC, TRITC, and Cy5 channels were separated as pictures with a color bar. The fluorescence-photomicrographs presented the diameters (μm) of CTC and a representative WBC and their respective DAPI stained nucleus. The immunocytochemistry-photomicrographs are presented with a scale bar, magnification information, digital reticule, as well as the diameters (μm) of CTC and a representative WBC.

**Figure 4:**
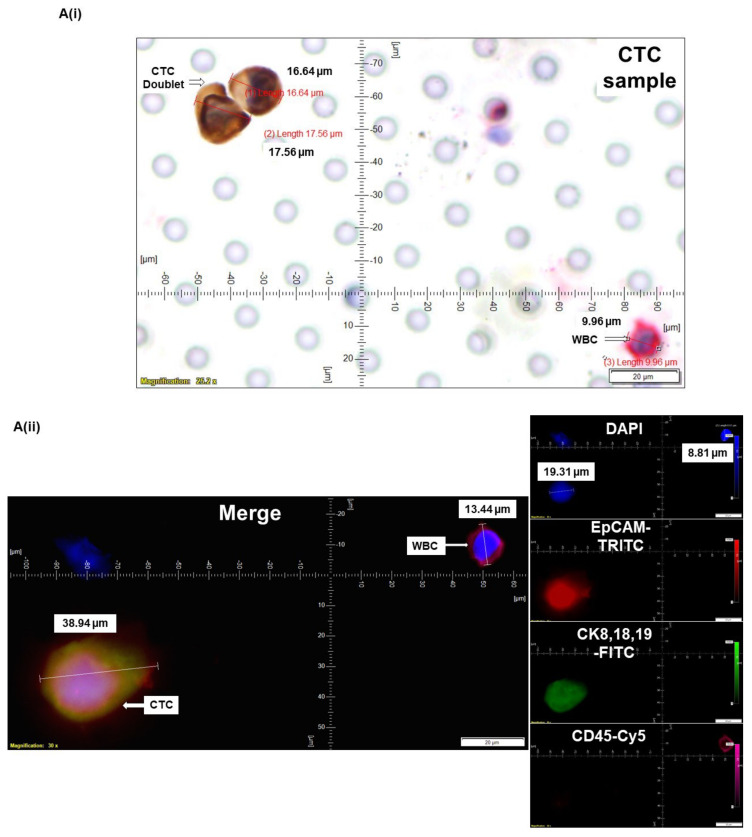

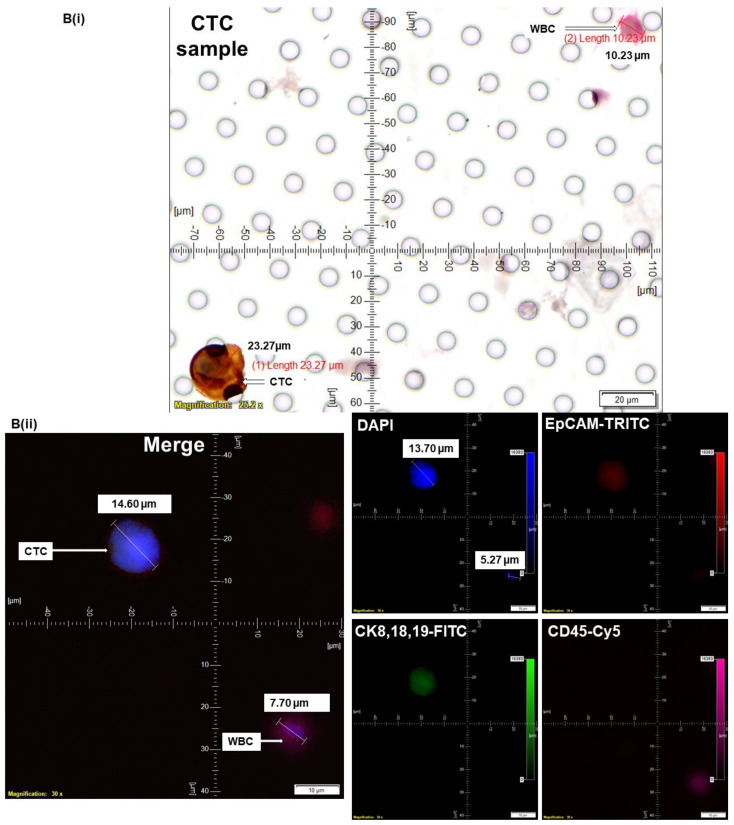
Determining CTC by ICC×2 in endometrial and ovarian cancers: CTCs were captured from blood samples from patients with endometrial (**A**) and ovarian (**B**) tumors and enumerated using ICC×2 (**Ai**,**Bi**). Blood samples were spiked (Spiked samples) with titrating number (250 cells/100 cells) of NCI-H441 cells separately for ICC×2. Corresponding CTC enumeration by IF×3 (**Aii**,**Bii**) is presented. For IF×3, pictures were taken at 60× oil objective of an Olympus IX71 Microscope with DAPI/FITC/TRITC/CY5 filter sets. For ICC×2, pictures were taken at 40× objective of an Olympus BX43 Microscope. The magnification, scale bar, and digital reticle are represented for each photomicrograph. Fluorescence images from DAPI, FITC, TRITC, and Cy5 channels were separated as pictures with a color bar. The fluorescence-photomicrographs presented the diameters (μm) of CTC and a representative WBC and their respective DAPI stained nucleus. The immunocytochemistry-photomicrographs are presented with a scale bar, magnification information, digital reticule, as well as the diameters (μm) of CTC and a representative WBC.

**Figure 5:**
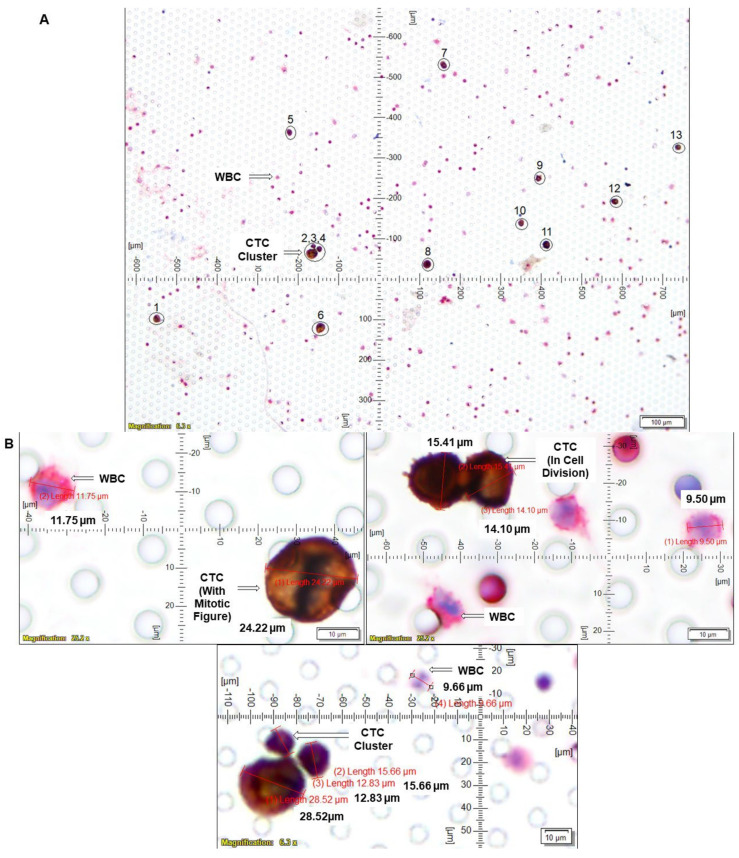
Clinical relevance of determination of the number of CTCs using a single case study: we determined CTC by ICC×2 from the blood of a patient with grade 2 stage I endometrial cancer: CTCs were captured from blood samples from the patient and enumerated using ICC×2 (**A**,**B**). Blood samples were spiked (Spiked samples) with titrating number (250 cells/100 cells) of NCI-H441 cells separately for ICC×2. For ICC×2, pictures were taken at 40× objective of an Olympus BX43 Microscope. The magnification, scale bar, and digital reticle are represented for each photomicrograph. We recorded up to 100 CTCs in the 7.5 mL of the blood with 13 CTCs in a single microscopic field (**A**) and mitotic CTCs with a mitotic figure and a cluster of 3 CTCs (**B**). The immunocytochemistry-photomicrographs presented with a scale bar, magnification information, digital reticule, as well as the diameters (μm) of CTC and a representative WBC.

CellSieve enumeration kit from Creatv Microtech was used for CTC detection employing standard triple immunofluorescent (IF×3) staining [21,22,23] with certain modifications. In short, 7.5 mL whole blood and 7.5 mL fixation buffer were mixed gently in a 50 mL conical tube and incubated for 15 min at room temperature. The filter holder containing the membrane with a 7-micron pore size was assembled during this incubation period. KD scientific Legato 110 syringe pump was used to draw fluid through the filter (‘push’ program; 60% force) to move PBS up through the filter to pre-wet it. Next, the fixed blood sample was applied to the filter and pulled through. As per the manufacturer’s protocol, we used a kit with CK8,18,19-FITC, EpCAM-PE, and CD45-Cy5 for the staining of CTCs. The images were acquired using Olympus cellSens 1.18 LIFE SCIENCE IMAGING SOFTWARE (OLYMPUS CORPORATION). We used the principle of CD45^−^/CK8,18,19^+^/EpCAM^+^/DAPI for our immuno-fluorescence method. DAPI was used for the evaluation of the nuclear size and morphology. In all the photomicrographs of figures (Figure 1, Figure 2, Figure 3, Figure 4 and Figure 5), we indicate the measurement of the nuclear diameters.

### 2.6. Validation of CTC Assays by Double Immuno-Cytochemistry Assay and Parallel Triple Immunofluorescence Assays

Parallel identification of CTCs by triple-immunofluorescence assay was performed to validate ICC×2. Spike samples of tumor cell lines from endometrial, ovarian, breast, and lung cancers were used. The cell lines were prefixed, and the number of cells in the sample was titrated down (100 cells per spike) to test the sensitivity. The specificity was tested using epithelial cancer cell lines compared to CD31-positive HUVEC cells or normal Human Uterine Fibroblasts (HUF). Leucopak, PBMC, and blood (age-matched) from otherwise healthy persons were used to test the absence of CTC in normal individuals. The test samples were run parallel to spiked samples each time as an internal positive control. The background autofluorescence for all five channels (Microscope Olympus IX71 with DAPI/FITC/TRITC/CY5 filter sets) was tested in both CTC samples as well as spiked samples. The test samples were stained similarly except without the cocktail of primary antibody-conjugate(s). We used the same blood sample twice and separately spiked it with NCI-H441 and HUVEC cells to test the cross-reactivity between epithelial cells and endothelial cells in the peripheral blood. The spiked blood samples were stained with CD31 Kit (containing antibody cocktail for CK 8,18,19/CD45/CD31; specific for detecting endothelial cells) and EpCAM Kit (having antibody cocktail for CK 8,18,19/CD45/EpCAM; specific for detecting epithelial cells). Pictures were taken at 60× oil objective of an Olympus IX71 Microscope with DAPI/FITC/TRITC/CY5 filter sets. The image was acquired using Olympus cellSens 1.18 LIFE SCIENCE IMAGING SOFTWARE (OLYMPUS CORPORATION). The validation of the double-immunocytochemistry assay was based on a parallel validation of the triple immunofluorescence assays in the same blood samples. We used tumor cells from different organ-type cancers for validation. The expression of proteins (CK 8,18,19^+^/EpCAM^+^/CD45^−^/SMA^−^/CD31^−^) was simultaneously and independently tested using immunocytochemistry, immunofluorescence, and flow cytometry. Once validated, we ran blood samples by immunocytochemistry and immunofluorescence. Out of our 91 blood samples used for the study, we determined CTC by immunofluorescence in 89 blood samples and by immunocytochemistry in 47 blood samples. We used both immunofluorescence and immunocytochemistry methods in 44 blood samples for the concordance study. Each time a blood sample was run (immunocytochemistry and immunofluorescence), we simultaneously ran a tumor cell line, NCI-H441, with it as a positive control. A presentative picture of the NCI-H441 tumor cell line (CK 8,18,19^+^/EpCAM^+^/CD45^−^/DAPI) as positive control is presented in the figures (Figure 2 and Figure 3).

## 3. Results

A total of 91 patients were enrolled in the study (informed consent), and their blood samples were received for standardization and detection of CTC (Table 1). Table 2 presents the background characteristics of the patients. Table 3 presents patients’ pre-treatment status at surgery and history of other cancers. Among the blood samples received from 71 patients with endometrial carcinomas (used for the standardization and testing of CTC), we observed endometrioid carcinoma (invasive and non-invasive) as the predominant pathologic subtypes of the disease. The rest of the subtypes included carcinosarcoma and mixed endometrial adenocarcinomas. Among the blood samples received from 11 patients with ovarian carcinomas (used for the standardization and testing of CTC), we observed serous carcinoma (low and high grades) as the predominant pathologic subtypes of the disease. The rest of the subtypes included ovarian adenocarcinoma, adult granulosa cell tumors, ovarian mucinous cystadenoma, and appendiceal mucinous neoplasms. The different pathological subtypes of the lung disease in patients from whom we received our blood samples included squamous cell carcinoma, well-differentiated neuroendocrine tumors, and invasive adenocarcinomas. Our study included 48% of patients with Grade 1 disease, out of which blood samples of 6 patients were used for standardization and 38 were used for CTC-testing. Sixteen percent of the total patients had Grade 2 disease, out of which blood samples of 4 patients were used for standardization, and 11 were used for CTC-testing. Eighteen percent of the total patients had Grade 3 disease, out of which blood samples of 3 patients were used for standardization, and 14 were used for CTC-testing. We first standardized CTC detection by IF×3 by standard triple-immunofluorescence protocol [21,22,23] using blood from patients’ samples spiked with several tumor cell lines, breast, lung, endometrial and ovarian cancers (Figure 1). A total of 15 blood samples from patients with cancer of different organ types were used for standardization (IF×3 and ICC×2). In addition to the blood from patients’ samples, parallel blood samples from age-matched healthy individuals’ leucopaks and PBMCs were used for standardization and testing auto-fluorescence. Once IF×3 was standardized, we validated our novel procedure of ICC×2-based CTC determination using standard IF×3.

### 3.1. Standardization and Validation of CTC by IF×3 Using Breast, Ovarian, and Lung Cancer Cell Lines

Patients’ blood samples were spiked with titrating numbers (1000 cells, 750 cells, 375 cells, 250 cells/100 cells) of MCF7, OVCAR3, HCC1975, and NCI-H441 tumor cell lines. The captured MCF7 cells, which were used to spike blood samples, were stained with either DAPI/CK-FITC/EpCAM-PE/CD45-Cy5 or DAPI/CK-FITC/CD31-PE/CD45-Cy5. When stained with DAPI/CK-FITC/EpCAM-PE/CD45-Cy5, the MCF7 cells were found to have a proportionately higher diameter (size 15–17 μm) bearing the typical salt-pepper nuclear morphology in a DAPI stain. The cytoplasm of the cells was positive for CK, 8,18,19, and EpCAM. When stained using the DAPI/CK-FITC/CD31-PE/CD45-Cy5 kit, the MCF7 cells were CK8,18,19^+^/CD31^−^/CD45^−^/DAPI^+^ (Figure 1(Aii)) as compared with CK8,18,19^+^/EpCAM^+^/CD45^−^/DAPI^+^ when stained using the DAPI/CK-FITC/EpCAM-PE/CD45-Cy5 antibodies (Figure 1(Ai)). A similar pattern of stains (CK8,18,19^+^/EpCAM^+^/CD45^−^/DAPI^+^) was observed for OVCAR3 (Figure 1B), HCC1975 (Figure 1C), and NCI-H441 (Figure 1D) cells using the DAPI/CK-FITC/EpCAM-PE/CD45-Cy5 antibodies. Since we did not have the confocal images, we could identify the plasma-membrane EpCAM positivity of a tumor cell depending on the orientation of the cell on the microfilter as shown in HCC1975 (Figure 1C) and NCI-H441 (Figure 1D) cells using the DAPI/CK-FITC/EpCAM-PE/CD45-Cy5 antibodies. All cell lines were found as negative for CD45-Cy5 for both sets of antibody cocktails.

### 3.2. Validation Spectrum of CTC by IF×3 Using Blood from Patients with Different Clinical Statuses, and Sample Origin

We validated CTC by IF×3 from a spectrum of blood from patients with different (Figure 2A) clinical status, Grade 1, Stage IA nonmetastatic endometrial cancers (pT1a pN0) (Figure 2(Ai)) and Grade 3, Stage IVB metastatic (pT3a N0 M1) (Figure 2(Aii)) in endometrial cancers, and (B) samples of origin, including biopsy sample from a liver lesion in metastatic squamous cell carcinoma (Figure 2(Bi)) and during surgical resection of Grade 1 (pT1b N0) tumor in lung cancers (Figure 2(Bii)) using the DAPI/CK-FITC/EpCAM-PE/CD45-Cy5 antibody cocktail. We used blood from the patients with metastatic disease as an internal positive control for the presence of CTC. Confirming the standard IF×3 protocol, we observed that CTCs in each of the above-mentioned samples were more than 15–20 micron in size with an evident pathological/morphological nuclear characteristic of a tumor cell (a nuclear/cytosol ratio > 50%) by DAPI and were CK8,18,19^+^/EpCAM^+^/CD45^−^/DAPI^+^ when stained using the DAPI/CK-FITC/EpCAM-PE/CD45-Cy5 antibodies.

### 3.3. Standardization and Validation of CTC by ICC×2 in Reference to Spiked IF×3 in Endometrial and Ovarian Cancers

Having confirmed the determination of CTC by IF×3 in a spectrum of blood samples, we standardized the CTC by ICC×2 (CK8,18^+^/CD45^−^). We validated ICC×2 with spiked control using parallel IF×3 and ICC×2 procedures in the same blood sample in endometrial and ovarian cancers (Figure 3). As presented before, CTCs were captured from blood samples from patients with endometrial (Figure 3A) and ovarian (Figure 3B) tumors and enumerated using ICC×2 (Figure 3(Ai,Bi)) in reference to IF×3 (Figure 3(Aii,Bii)). Blood samples were spiked (Spiked samples) with titrating numbers (250 cells/100 cells) of NCI-H441 cells separately for both ICC×2 and IF×3. Both CTC and spiked samples exhibited a similar pattern of cell size and staining pattern (CK8,18^+^/CD45^−^) by ICC×2, which was comparable to the corresponding IF×3 staining patterns. We observed a cluster of CTCs with different diameters similar to the spiked samples of NCI-H441 (Figure 3(Ai)). CTCs were characterized and distinguished by their diameter(s), nuclear morphology (a nuclear/cytosol ratio >50%), and CK8,18^+^/CD45^−^ staining. In contrast, WBCs were smaller in size (9–15 μm) with their characteristics of nuclear morphology and CK8,18^−^/CD45^+^ staining.

### 3.4. Determining CTC by ICC×2 in Endometrial and Ovarian Cancers

Having established ICC×2 staining validated using parallel IF×3 spiked with tumor cell lines in blood samples of different solid tumors, we finally tested the method for the determination of CTC by ICC×2 and validated it with corresponding CTC determination by IF×3. CTCs were captured from blood samples from patients with Grade 1 Stage IA (pT1a pN0 (sn)) endometrial (Figure 4A) and Grade 1 Stage IVA (pTIVb pN0 pM1b) ovarian (Figure 4B) tumors and enumerated using ICC×2 (Figure 4(Ai,Bi)). In line with the earlier results, the CTC in ICC×2 were CK8,18^+^/CD45^−^ while the WBCs were CK8,18^−^/CD45^+^ in ICC×2, which matched with the IF×3 validation samples where CTCs were larger in diameter (>15–20 μM) with CK8,18,19^+^/EpCAM^+^/CD45^−^/DAPI^+^ while WBCs were smaller in diameter (9–15 μm) with CK8,18,19^−^/EpCAM^−^/CD45^+^/DAPI^+^ (Figure 4(Aii,Bii)).

Table 1 shows that our study included 63% of patients with Stage I disease, out of which blood samples of 6 patients were used for standardization and 51 were used for CTC-testing. Five percent of the total patients had Stage II disease, out of which a blood sample of one patient was used for standardization, and four were used for CTC-testing. Fourteen percent of the total patients had Stage III disease, out of which blood samples of 3 patients were used for standardization, and 10 were used for CTC-testing. Ten percentof our enrolled patients had Stage IV metastatic disease, out of which blood samples of four patients were used for standardization while the remaining five were used for CTC-testing. Although the percentage of CTC-positive patients with Stage I, Stage II, and Stage III diseases were 45%, 50%, and 30%, respectively, the percentage of CTC-positive patients rose to 100% in the blood of patients with Stage IV metastatic diseases. We tested the sensitivity of the ICC method by titrating the number of spiked cells; 25 cells/test, 50 cells/test, and 100 cells/test. The recovery was >50% for 25 cells/test, >60% for 50 cells/test, and >65% for 100 cells/test. The specificity was tested by CD45^−^/CK8,18,19^+^/EpCAM^+^/DAPI stain for nuclear size and morphology. We also used cell lines from cancer of different organ types, namely endometrial, ovarian, breast, and lung. We also used the commercially available CD31-kit to demonstrate the fact that CTC/tumor cells are CD31 negative (Figure 1) and to rule out a false positive. We used blood from donors, leucopaks, and PBMCs for the control.

Table 1 shows 45% CTC positivity in patients with Stage I disease. However, a detailed interrogation of the result revealed that the high percentage (45%) was obtained because we calculated the “presence of CTC” recorded in a “yes-or-no format.” Importantly, we observed that out of 54 patients (those we tested for CTC) with Stage I endometrial disease, 28 patients were CTC-negative, and 26 were CTC-positive. Out of 26 CTC-positive patients, 77% (20/26) had <1–3 CTCs.

We could not determine any statistically significant correlation between grades and the number of CTC as the numbers of patients with high-grade tumors in our study cohort were significantly lower than the numbers of patients with low-grade tumors. Table 4 presents the Grade-wise distribution of patients’ blood samples used for the standardization and testing of CTCs, along with tumors from each pathology.

However, we observed an interesting association between the presence of CTC and the high grade/stage of the disease. Out of a total of nine patients with Stage IV/Metastatic disease, blood samples from three patients were used for standardization. Of six patients whose blood samples were used for CTC detection, 100% tested positive for CTCs. With regard to High-Grade (Grade 3) patients, we had a total of 18 patients with Grade 3 disease. Of these patients, blood samples from two patients were used for standardization. Of the remaining 16 patients, a 69% CTC positivity (11/16) was observed. There were four patients who were diagnosed with both Grade 3 and Stage IV/Metastatic disease. The blood samples from one of these patients were used for standardization; out of the remaining three patients with both Grade3 and Stage IV/Metastatic disease, 100% were tested and were found to have CTCs.

Since the CTC expression varied depending on the Stage and the Grade of the disease, we did not consider the median or average expression values across all; we stratified patients with CTC positivity according to the Stage and the most common histology type, endometrioid adenocarcinoma.

However, we determined the rate of detection of CTC in endometrial cancers. In endometrial cancers, the detection rate was 55% (35/64). The rate of detection can be explained by the fact that 75% (48/64) of our CTC-tested patients were Stage I.

We also tested the CTC detection rate in the most common histological type of endometrial cancer, endometrioid adenocarcinoma. Out of 64 patient samples tested for CTC, 42 patients had endometrioid adenocarcinoma (Out of 42, 86% were Stage I; 36/42), and the CTC detection rate was 60% (25/42). Out of 25, 80% had Stage I disease (20/25). Interestingly, 76% (19/25) presented with 1–3 CTCs counts; out of these 19, 79% had Stage I disease (15/19).

We tested the clinical relevance of a high number of CTCs in a single case study. The presence of >100 CTCs (Figure 5) was observed at the surgery in a patient with Grade 2, stage IA endometrioid adenocarcinoma, 6% MI, and absence of lymphovascular invasion, absence of LN Status as well as Uterine Serosa and Cervical Stroma involvement. We observed 13 CTCs in a microscopic field with mitotic figures as well as 3-cell CTC clusters. The patient received four fractions of HDR vaginal cuff brachytherapy. The patient came in for surveillance, and a lesion was observed. Biopsy demonstrated recurrent endometrioid adenocarcinoma. A CT scan of the chest, abdomen, and pelvis revealed an area of poorly defined but somewhat mass-like enhancement in the region of the right vaginal cuff suspicious of disease recurrence. There were no other changes concerning additional metastatic disease elsewhere. The patient had an event within 6 months of the date of surgery.

## 4. Discussion

Our method of detection of CTC followed the standard CTC determination criteria including, (1) negative reactivity to immune cell marker (CD45), (2) positive reactivity to cytokeratin 8, 18, 19, (3) positive reactivity to EpCAM surface marker, and (4) morphologic characteristics [25]. Our method of determining CTC by ICC×2 gave us a *parallel double-detection format* (ICC×2 and IF×3) for a foolproof test with a higher confidence level in terms of specificity and sensitivity. We observed a concordance close to 80% in our cohort. We carried out the IF and ICC evaluation of CTC independent/without knowledge of the final pathology findings of these specimens; however, such findings were incorporated after completing our IF/ICC of CTC data collections. The sensitivity of our method of employing *a parallel double-detection format* was also tested in the built-in nature of our patient cohort. Close to 65% of our blood samples for CTC detection (standardization and testing) were samples drawn from patients with Grades 1 and 2 diseases. Table 1 showed that 68% of our blood samples for CTC detection (standardization and testing) were samples drawn from patients with Stage I and II diseases, wherein we were able to detect the presence of CTC (Table 1). Interestingly, 45% and 50% of patients with Stage I and II diseases tested positive for CTC, respectively, indicating the strength of the method and the format of determination. *Our testing format can thus be utilized in monitoring the progression of the disease post-surgery or in an adjuvant setting, providing a valuable indicator of the metastatic potential* via *longitudinal CTC detection.* As expected, 100% of our patients with Stage IV metastatic disease tested positive for CTC, which can be viewed as a positive control within a disease population. Thus, our method is built on strong validation data, including internal validation, technical validation, and disease-population-based positive and negative validation controls. We also tested CTC in blood samples from patients undergoing both biopsies and surgeries.

Studies reported the feasibility of detection of CTCs using isolation by size-based Epithelial/Trophoblastic Tumor cells (ISET^®^) filters and stain by May–Grünwald–Giemsa in conjunction with identification criteria of nuclear irregularity, negative reactivity to immune cell marker as well as endothelial cell markers, and presentation of visible cytoplasm [26]. To test the negativity of CTC for CD31 in IF×3, we used the additional staining kit for DAPI/CK-FITC/CD31-PE/CD45-Cy5. HUVEC (positive control for CD31 and negative control EpCAM) and NCI-H441 (positive control for EpCAM and negative control for CD31) cells as validation controls. We used spiked HUVEC cells to represent the cross-reactivity of the probable endothelial cells in the blood. CK8,18,19^−^/EpCAM^−^/CD45^+^/DAPI^+^ WBCs were CK8,18,19^−^/CD31^−^/CD45^+^/DAPI^+^. CK8,18,19^+^/EpCAM^+^/CD45^−^/DAPI^+^ NCI-H441 cells were CK8,18,19^+^/CD31^−^/CD45^−^/DAPI^+^. HUVEC cells were CK8,18,19^±^/CD31^+^/CD45^−^/DAPI^+^.

Our *parallel double-detection format* for CTC determination is efficient as it can be ready for pathological evaluation within the standard working hours of one day. The procedure is laboratory friendly and requires basic equipment and microscopes, and can be carried out with a standard grad-school laboratory setup compared with the FDA-approved CellSearch semi-automated CTC detection system or the CTC detection sensitivity of ISET [26] or using an immunomagnetic enrichment [25]. Hence, the method is cost-effective, and the cost of the consumables per 7.5 mL blood sample can be estimated at around $500 only. Thus our method can be performed at a comprehensive cancer center as well as at a community-based small cancer hospital with limited resources. Since we did not compare the method with the rest of the available methods for CTC enumeration, the data for the comparison are currently unavailable. Yet the method has its niche and edge for the above reasons. Although our *parallel double-detection format* for the determination of CTC is limited to at least 16 mL of blood, the method compensates the volume of blood for the sensitivity and specificity of CTC. However, the main trade-off for this method is its limited capacity to scale in a demanding, high-throughput situation.

One of the established pathological parameters associated with the prognosis is the presence or absence of LVSI (Lympho-Vascular Space Invasion). Our method of CTC determination will quickly provide a unique opportunity to interrogate CTC’s role as a more sensitive risk factor vis-à-vis standard pathological parameters like LVSI in the context of particular histology, grades, and Stage of the disease. This might provide an opportunity to study wherein CTC can be used preoperatively (after malignant solid tumors are diagnosed on biopsies) as risk stratification for sentinel lymph nodes (SLN).

Cell-free (cf) circulating tumor (ct) derived DNA is released from tumor cells into the circulation and is often detected as part of routine liquid biopsy compared to CTC for clinical decision making. The ctDNA is used as (1) direct detection of early-stage cancers, (2) a marker for the detection of minimum residual disease, (3) an important tool to provide prognostic information, and (4) as an indicator of drug response in non-invasive liquid biopsies [27]. However, the critical challenge of this type of liquid biopsy has been in the detection/characterization of small amounts of ctDNA in large populations of cfDNA, as these analyses need to distinguish ctDNA alterations from cfDNA variants related to clonal hematopoiesis [28]. Blood-based deep-sequencing often encounters concerns about detection and misclassification of white blood cell (WBC)-derived variants in cfDNA associated with clonal hematopoiesis, especially in older patients [29,30]. In fact, Hu et al. reported a false-positive plasma genotyping due to clonal hematopoiesis where most JAK2 mutations, some TP53 mutations, and rare KRAS mutations detected in cfDNA were derived from clonal hematopoiesis instead of the tumor as mutations detected in plasma, particularly in genes mutated in clonal hematopoiesis, which might not represent the true tumor genotype, the study concluded [31]. The detection of non-tumor-derived clonal hematopoietic mutations (TP53, DNMT3A, etc.) has been reported as a source of the biological background noise of ctDNA detection that could lead to an inappropriate therapeutic decision.

The power of a longitudinal CTC, which enables serial assessments at multiple time points along a patient’s journey, during or after surgery/treatment, is undeniable. However, a recent article by Vasseur et al. delineated the limitations of using CTC data in routine clinical practice [3]. In the view of currently published or ongoing trials assessing the clinical utility of CTCs [3], it can be recognized that there exist challenges in the enumeration and phenotyping of CTC [19]. The limitations of CTCs in clinical practice are (1) the low detection rate with currently available techniques [3] and (2) the need for a costly comprehensive laboratory setup. Cost-effectiveness, yet specific, sensitive, and fail-safe nature of our laboratory friendly method of CTC enumeration will potentially support prospective studies with uniform and standardized definitions of CTCs that are urgently needed [17] to evaluate the full potential of CTCs not only as prognostic, predictive, and intermediate endpoint markers but also as PD biomarkers in the future. We are currently assessing the expression of PD-L1 in CTC, which may be helpful in considering the use of PD-1 inhibitors in clinical practice. The limitation of our platform is built in its development in a community-based cancer center; the platform is not yet tested in a prospective clinical trial. To this end, we are also actively pursuing customization of the antibody cocktail to profile the cancer-specific cell surface protein molecules (e.g., CA125) for future studies.

The strength of our method is built in its inherent development in a community-based cancer center; the method is cost-effective, time-sensitive, laboratory-friendly, and needs a single full-time employee. To this end, we tested the clinical relevance of our method in a case study. We reported on a stage I patient with >100 CTCs at surgery (with 13 CTCs in a single microscopic field; Figure 5). The patient with endometrioid adenocarcinoma had no apparent pathological features indicative of high risk for recurrence. Unfortunately, she presented with an adverse event within 6 months of surgery, strongly indicating the prognostic significance of CTC as reported in the earlier studies in different organ type cancers.

## 5. Conclusions

The need for easy detection of CTC is undeniable. Our user-friendly and cost-effective detection method provided an opportunity to incorporate CTC detection as a companion entity with the standard diagnostic and monitoring tests in clinics. The power of the method can be tested as a single-point and multi-point longitudinal mode in a clinical setting at the baseline, during, and after a treatment regimen. The baseline evaluation of CTC can be helpful for patient stratification, while longitudinal CTC evaluation during and after treatment can be useful for monitoring treatment response and early indicators of disease progression/drug resistance, respectively. The study presented in the MS is part of a patent application (United States Patent and Trademark Office; Application number 16/875,910.

## Figures and Tables

**Table 1:** Stage-wise distribution of patients’ blood samples used for the standardization and testing of CTCs, with tumors from each pathology.

Stages of Patients with Different Tumors (Endometrial, Ovary, Lung, Esophageal, Prostate, and Liver)	Total Percentage of Patients’ Blood Used for the Study (%, n = 91)		Number of Blood Samples Used for CTC Standardization (n = 91)		Number of Blood Samples Used for CTC Testing (n = 91)	Percentage of Patients with Positive CTC (IF and/or ICC) (%)
**Stage I**	**63%**		6		51	45%
**Stage II**	5%		1		4	50%
**Stage III**	14%		3		10	30%
**Stage IV (Metastatic)**	10%		4		5	100%
Tumors from Each Organ Type						
**Tumors from Each Pathology**	**Endometrial**	**Ovary**	**Lung**	**Esophageal**	**Prostate**	**Liver**
**Stage I**	54	3	0	0	0	0
**Stage II**	2	1	2	0	0	0
**Stage III**	9	3	0	1	0	0
**Stage IV (Metastatic)**	3	2	1	0	2	1

**Table 2:** Pathology parameters of organ type (endometrial, ovarian, lung, prostate, liver, and esophageal) tumors used for the study (LVI = Lymphovascular Invasion; MI = Myometrial Invasion; MSI = Microsatellite Instability; NA = Not Applicable; ND = Not Determined; NAV = Not Available).

De-Identified Patient Code	Pathological Parameters of Tumor Samples from Patients with Endometrial Cancer						
	Tumor Type-Histological	TMN	Grade	Stage	LVI	MI (%)	MSI
**CTC-EC-691**	Endometrioid adenocarcinoma	pT2 pN0	1	II	Present	25	NAV
**CTC-EC-702**	Endometrioid adenocarcinoma	pT1a N0 (sn)	1	IA	Absent	14	NAV
**CTC-EC-713**	Endometrioid adenocarcinoma	pT1a N0 (sn)	1	IA	Absent	15	NAV
**CTC-EC-724**	Endometrioid adenocarcinoma	pT1b N1a	1	IIIC1	Present	95	NAV
**CTC-EC-735**	Endometrioid adenocarcinoma	pT1a pN0 (i+) pMX	2	IA	Present	46	NAV
**CTC-EC-746**	Endometrioid adenocarcinoma	pT1a pN0	3	IA	Present	29	NAV
**CTC-EC-757**	Endometrioid adenocarcinoma	pT1a pNX	1	IA	Absent	0	NAV
**CTC-EC-768**	Endometrioid adenocarcinoma	pT1a pN0	1	IA	Absent	11	NAV
**CTC-EC-779**	Endometrioid adenocarcinoma	pT1b N1a	1	IIIC1	Present	67	NAV
**CTC-EC-7810**	High grade papillary serous carcinoma	pT3b pNX	3	IIIB	Absent	100	NAV
**CTC-EC-7911**	Endometrioid adenocarcinoma	pT1a NX	1	IA	Absent	9	NAV
**CTC-EC-8012**	Endometrioid adenocarcinoma	pT1apN0(sn)	1	IA	Absent	14	NAV
**CTC-EC-8113**	Endometrioid adenocarcinoma	pT1a pN0 (sn)	1	IA	Absent	14	NAV
**CTC-EC-8214**	Extensive mutltifocal complex hyperplasia with atypia	NA	ND	I	ND	ND	NAV
**CTC-EC-8315**	Residual carcinosarcoma	pT1a pN0	ND	IA	Absent	26	NAV
**CTC-EC-8416**	Endometrioid adenocarcinoma	pT1a pNX	1	I	Absent	28	NAV
**CTC-EC-8517**	Endometrioid adenocarcinoma	pT2 NX	2	II	Present	87	NAV
**CTC-EC-8618**	Carcinosarcoma	pT2 N1mi	3	IIIC1	Present	72	Stable
**CTC-EC-8719**	Endometrioid adenocarcinoma	pT1a pN0sn	1	IA	Absent	0	NAV
**CTC-EC-8820**	Endometrioid adenocarcinoma	pT1a pN0sn	1	IA	Absent	0	High
**CTC-EC-8921**	Carcinosarcoma with high grade serous carcinoma and rhabdomyosarcomatous differentiation	pT1a N1mi	3	IIIC1	Absent	38	Stable
**CTC-EC-9022**	Endometrioid adenocarcinoma (metastatic)	pT3b pNX pM1	3	IV	Absent	50	Stable
**CTC-EC-9223**	Endometrioid adenocarcinoma	pT1a N0	2	IA	Absent	44	NAV
**CTC-EC-9324**	Benign endometrial polyp	NA	NA	NA	NA	NA	NAV
**CTC-EC-9525**	Endometrioid adenocarcinoma with squamous cell differentiation	pT1a N0	1	IA	Absent	0	NAV
**CTC-EC-9626**	Endometrioid adenocarcinoma	pT1a N0	1	I	Absent	0	NAV
**CTC-EC-9727**	Endometrioid adenocarcinoma	pT1a pN0	1	IA	ND	25	NAV
**CTC-EC-9828**	Endometrioid adenocarcinoma	pT1a N0(i+)	1	I	Absent	17	NAV
**CTC-EC-9929**	Endometrioid adenocarcinoma	pT1b N0	3	IB	Absent	95	NAV
**CTC-EC-10030**	Benign endometrial polyp	NA	NA	NA	NA	NA	NAV
**CTC-EC-10131**	Endometrioid adenocarcinoma	pT1a N0	2	I	Absent	11	High
**CTC-EC-10232**	Endometrioid adenocarcinoma	pT1a N0	1	IA	Absent	29	NAV
**CTC-EC-10333**	Endometrioid adenocarcinoma	pT1a (sn) pN0 pMX	3	IA	Absent	43	NAV
**CTC-EC-10434**	Endometrioid adenocarcinoma	pT1a N0	1	IA	Present (?)	36	NAV
**CTC-EC-10535**	Complex atypical hyperplasia	NA	NA	NA	NA	NA	NAV
**CTC-EC-10636**	Endometrioid adenocarcinoma	pT1a N0	1	IA	Absent	17	NAV
**CTC-EC-10737**	Endometrioid adenocarcinoma	pT1a N0	1	IA	Absent	34	NAV
**CTC-EC-10838**	Endometrioid adenocarcinoma	pT1a (sn) N0	1	IA	Absent	13	NAV
**CTC-EC-10939**	Endometrioid adenocarcinoma	pT1a	2	IA	Present	25	NAV
**CTC-EC-11040**	Endometrioid adenocarcinoma	pT1a N0	1	IA	Absent	6	NAV
**CTC-EC-11141**	Endometrioid adenocarcinoma	pT1a pN0	3	IA	Absent	37	NAV
**CTC-EC-11242**	Endometrioid adenocarcinoma	pT1a (sn) N0	1	IA	Absent	35	NAV
**CTC-EC-11343**	Endometrioid adenocarcinoma	pT1a N0	1	IA	Absent	< 50%	NAV
**CTC-EC-11444**	Endometrioid adenocarcinoma	pT1b N0	3	IB	Absent	90	NAV
**CTC-EC-11545**	Endometrioid adenocarcinoma	pT1a pN0 (i+) (sn)	2	IA	Absent	15	NAV
**CTC-EC-11646**	Endometrioid adenocarcinoma	pT1a N0	2	IA	Absent	32	NAV
**CTC-EC-11747**	Endometrioid adenocarcinoma	pT1a N0	2	IA	Absent	8	High
**CTC-EC-11848**	High-grade serous endometrial adenocarcinoma	pT1a N2mi	3	IIIC2	Present	46	NAV
**CTC-EC-11949**	Endometrioid adenocarcinoma	pT1a sn N0	1	IA	Absent	0	NAV
**CTC-EC-12050**	Endometrioid adenocarcinoma	pT1b sn N1a	2	IIIC1	Present	57	NAV
**CTC-EC-12151**	Endometrioid adenocarcinoma	pT1a pN0 (sn)	1	IA	Absent	22	NAV
**CTC-EC-12252**	Endometrioid adenocarcinoma	pT1a (sn) pN0	1	IA	Absent	19	High
**CTC-EC-12353**	Endometrioid adenocarcinoma	pT1a pN0	1	IA	Absent	30	NAV
**CTC-EC-12454**	Endometrioid adenocarcinoma	pT1a pN0 (sn)	1	IA	Absent	38	NAV
**CTC-EC-12555**	Endometrioid adenocarcinoma	pT1a pN0	1	IA	Absent	0	NAV
**CTC-EC-12656**	Endometrioid carcinoma	pT1a pNX pMX	1	IA	Absent	0	NAV
**CTC-EC-12757**	Endometrioid adenocarcinoma	pT1a (sn) pN0	1	IA	Absent	41	NAV
**CTC-EC-12858**	Endometrioid adenocarcinoma	pT1a N0	1	IA	Absent	23	NAV
**CTC-EC-12959**	High-grade serous endometrial adenocarcinoma	pT2 (sn) N2mi	3	IIIC2	Present	87	NAV
**CTC-EC-13060**	Mixed cell adenocarcinoma, (50% high-grade serous, 50% clear cell)	pT1a N0 M1	3	IVB	Absent	0	NAV
**CTC-EC-13161**	High-grade serous endometrial adenocarcinoma	pT3a (sn) pN0 (i+)	3	IVB	Present	0	NAV
**CTC-EC-13262**	Uterine carcinosarcoma	pT1a pN0	ND	IA	Absent	13	NAV
**CTC-EC-13363**	Mixed cell adenocarcinoma, (10% high-grade serous carcinoma, 90% endometrioid)	pT1a pN0 (sn)	3	IA	Absent	13	NAV
**CTC-EC-13464**	Endometrioid adenocarcinoma	pT1a (sn) N0	2	IA	Absent	6	NAV
**CTC-EC-13565**	Mixed cell adenocarcinoma, (90% high-grade serous, 10% endometrioid adenocarcinoma)	pT1a N0	3	IA	Absent	38	NAV
**CTC-EC-13866**	Endometrioid adenocarcinoma	pT1b N0(sn)	1	IB	Absent	64	High
**CTC-EC-14067**	Endometrioid adenocarcinoma	pT1a pNX	1	IA	Absent	35	NAV
**CTC-EC-14268**	Endometrioid adenocarcinoma	pT1a (sn) N0	2	IA	Absent	10	NAV
**CTC-EC-14369**	Endometrioid adenocarcinoma	pT1a pN1mi (sn)	1	IIIC1	Absent	46	NAV
**CTC-EC-14570**	Endometrioid adenocarcinoma	pT1a pN0 (sn)	2	I	Absent	25	NAV
**CTC-EC-14771**	Carcinosarcoma (predominantly endometrioid adenocarcinoma)	pT1a (sn) pN0i+	3	IA	Present	48	NAV
**De-Identified Patient Code**	**Pathological Parameters of Tumor Samples from Patients with Ovarian Cancer**						
	**Tumor Type—Histological**	**TMN**	**Grade**	**Stage**	**LVI**	**MSI**	
**CTC-OC-911**	Adenocarcinoma consistent with history of ovarian carcinoma	ND	ND	IIIC/IV	NA	Stable	
**CTC-OC-942**	Serous carcinoma	(y)pT3c pNX pMX	1	IIIC	Present	ND	
**CTC-OC-1363**	Adult granulosa cell tumor	pT1a NX	NA	IA	Absent	NAV	
**CTC-OC-1374**	Low grade serous carcinoma with abundant psammoma bodies (omentum)	ND	1	IIIA2	NA	Stable	
**CTC-OC-1395**	High-grade serous carcinoma	pT3b pN0	3	IIB	Absent	NAV	
**CTC-OC-1416**	Ovarian mucinous cystadenoma	NA	NA	NA	Absent	NAV	
**CTC-OC-1447**	Low grade serous borderline tumor with psammoma bodies	(m) pT3a pNX	1	IIIA	NA	NAV	
**CTC-OC-1468**	Simple cyst with giant cell reaction in the cyst wall	NA	NA	NA	Absent	NAV	
**CTC-OC-1489**	Low-grade appendiceal mucinous neoplasm	pT4b pN0 pM1b	1	IVA	Absent	NAV	
**CTC-OC-14910**	Low-grade serous carcinoma	pT1b pNX	1	IB	Absent	NAV	
**CTC-OC-15011**	Mucinous borderline tumor	pT1a	NA	1A	NA	NAV	
**De-Identified Patient Code**	**Pathological Parameters of Tumor Samples from Patients with Lung Cancer**						
	**Tumor Type—Histological**	**TMN**	**Grade**	**Stage**	**LVI**	**MSI**	
**CTC-LC-W201**	Moderately differentiated keratinizing squamous cell carcinoma	pT1c NX	2	IVC	Present	NAV	
**CTC-LC-W212**	Well differentiated neuroendocrine tumor (typical carcinoid)	pT1b pN0	1	ND	Absent	NAV	
**CTC-LC-W223**	Invasive moderately differentiated adenocarcinoma, multifocal	pT3 N0	2	IIB	Absent	NAV	
**CTC-LC-W234**	Necrotizing granulomatous inflammation	NA	NA	NA	NA	NAV	
**CTC-LC-W245**	Squamous cell carcinoma, moderately differentiated	pT3 N0 M0	2	IIB	Absent	NAV	
**De-Identified Patient Code**	**Pathological Parameters of Tumor Samples from Patients with Liver Neoplasm**						
	**Tumor Type—Histological**	**TMN**	**Grade**	**Stage**	**LVI**	**MSI**	
**CTC-LivC-R11**	Metastatic squamous cell carcinoma	NA	NA	NA	NA	Stable	
**De-Identified Patient Code**	**Pathological Parameters of Tumor Samples from Patients with Prostate Cancer**						
	**Tumor Type-Histological**	**TMN**	**Grade**	**Stage**	**LVI**	**MSI**	
**CTC-PC-M11**	Poorly differentiated adenocarcinoma	T3b N0 MX	3	IVB	Absent	NAV	
**CTC-PC-M22**	Metastatic adenocarcinoma of prostate	NA	NA	IVB	NA	Stable	
**De-Identified Patient Code**	**Pathological Parameters of Tumor Samples from Patients with Esophageal Cancer**						
	**Tumor Type—Histological**	**TMN**	**Grade**	**Stage**	**LVI**	**MSI**	
**CTC-EsoC-G11**	Esophageal adenocarcinoma	ypT3 N0	2	III	Present	NAV	

**Table 3:** Demographics of the patients whose blood samples were used for the study (F = Female; M = Male; BMI = Body Mass Index).

De-Identified Patient Code	Patient Demographics of Tumor Samples: Patients with Endometrial Cancer			
	Age at Surgery (Years)	Sex	BMI	History of Other Cancers/Pre-Treatment Status at Surgery
**CTC-EC-691**	65	F	41.3	None
**CTC-EC-702**	84	F	25.2	None
**CTC-EC-713**	79	F	41	None
**CTC-EC-724**	61	F	37.8	None
**CTC-EC-735**	64	F	41.2	None
**CTC-EC-746**	81	F	29	None
**CTC-EC-757**	49	F	44	None
**CTC-EC-768**	65	F	37.3	None
**CTC-EC-779**	60	F	28	None
**CTC-EC-7810**	68	F	34.9	None
**CTC-EC-7911**	56	F	60.1	None
**CTC-EC-8012**	76	F	30.1	History of breast cancer treated with chemotherapy approx. 40 years prior to diagnosis.
**CTC-EC-8113**	49	F	42.8	None
**CTC-EC-8214**	50	F	49.2	None
**CTC-EC-8315**	64	F	42.8	History of breast ductal carcinoma in situ two years prior to diagnosis, treated with anastrozole.
**CTC-EC-8416**	65	F	39.8	None
**CTC-EC-8517**	72	F	28.1	None
**CTC-EC-8618**	68	F	47	None
**CTC-EC-8719**	52	F	44.2	None
**CTC-EC-8820**	59	F	34.7	None
**CTC-EC-8921**	63	F	32.2	None
**CTC-EC-9022**	83	F	36.6	None
**CTC-EC-9223**	77	F	40.7	None
**CTC-EC-9324**	55	F	36.4	None
**CTC-EC-9525**	71	F	41.4	None
**CTC-EC-9626**	79	F	37.9	History of basal cell carcinoma of the skin. No chemo-treatment.
**CTC-EC-9727**	70	F	23.5	None
**CTC-EC-9828**	63	F	33.3	None
**CTC-EC-9929**	65	F	29.9	None
**CTC-EC-10030**	58	F	52.2	None
**CTC-EC-10131**	62	F	21.9	None
**CTC-EC-10232**	68	F	30.5	None
**CTC-EC-10333**	56	F	31.5	None
**CTC-EC-10434**	65	F	31.7	History of thyroid cancer
**CTC-EC-10535**	57	F	33.5	None
**CTC-EC-10636**	74	F	33.9	None
**CTC-EC-10737**	43	F	43.2	None
**CTC-EC-10838**	65	F	34.4	None
**CTC-EC-10939**	66	F	52	None
**CTC-EC-11040**	79	F	40.8	None
**CTC-EC-11141**	77	F	39.8	None
**CTC-EC-11242**	66	F	51.3	None
**CTC-EC-11343**	74	F	33.4	History of skin cancer
**CTC-EC-11444**	62	F	33.3	None
**CTC-EC-11545**	65	F	32.9	None
**CTC-EC-11646**	65	F	33.6	None
**CTC-EC-11747**	46	F	38.4	None
CTC-EC-11848 *	56	F	26.4	None
**CTC-EC-11949**	65	F	29.7	None
**CTC-EC-12050**	46	F	44.3	None
**CTC-EC-12151**	44	F	34.9	None
**CTC-EC-12252**	68	F	41.1	None
**CTC-EC-12353**	79	F	49.2	None
**CTC-EC-12454**	68	F	30.9	None
**CTC-EC-12555**	60	F	38.4	History of astrocytoma
**CTC-EC-12656**	62	F	43.9	None
**CTC-EC-12757**	71	F	35.6	None
**CTC-EC-12858**	71	F	53.3	None
**CTC-EC-12959**	67	F	44.3	None
**CTC-EC-13060**	84	F	35.5	None
**CTC-EC-13161**	59	F	35.2	None
**CTC-EC-13262**	68	F	33.1	None
**CTC-EC-13363**	62	F	31.8	None
**CTC-EC-13464**	75	F	26.9	History of skin cancer
**CTC-EC-13565**	60	F	62.7	None
**CTC-EC-13866**	70	F	35.2	None
**CTC-EC-14067**	71	F	48.1	None
**CTC-EC-14268**	73	F	37.4	None
**CTC-EC-14369**	68	F	31.6	None
**CTC-EC-14570**	74	F	34.3	None
**CTC-EC-14771**	53	F	27	None
**De-Identified Patient Code**	**Patient Demographics of Tumor Samples: Patients with Ovarian Cancer**			
	**Age at Surgery**	**Sex**	**BMI**	**History of Other Cancers/Pre-Treatment Status at Surgery**
**CTC-OC-911**	62	F	21.1	Heavily pre-treated with multiple chemotherapeutic agents
**CTC-OC-942**	58	F	28.9	None
**CTC-OC-1363**	52	F	32.3	None
**CTC-OC-1374**	58	F	42	None
**CTC-OC-1395**	62	F	28.3	None
**CTC-OC-1416**	44	F	28.5	None
**CTC-OC-1447**	64	F	47.3	None
**CTC-OC-1468**	79	F	25.3	History of Diffuse Large B-Cell Lymphoma treated with RCHOP
**CTC-OC-1489**	78	F	26.3	None
**CTC-OC-14910**	82	F	30.4	None
**CTC-OC-15011**	19	F	35.6	None
**De-Identified Patient Code**	**Patient Demographics of Tumor Samples: Patients with Lung Cancer**			
	**Age at Surgery**	**Sex**	**BMI**	**History of Other Cancers/Pre-Treatment Status at Surgery**
**CTC-LC-W201**	53	M	18.7	History of squamous cell carcinoma of lower lip treated with surgery
**CTC-LC-W212**	54	F	25.3	History of breast cancer
**CTC-LC-W223**	70	F	34.6	None
**CTC-LC-W234**	50	F	38.8	None
**CTC-LC-W245**	73	M	25.7	None
**De-Identified Patient Code**	**Patient Demographics of Tumor Samples: Patients with Liver Cancer**			
	**Age at Surgery**	**Sex**	**BMI**	**History of Other Cancers/Pre-Treatment Status at Surgery**
**CTC-LivC-R11**	66	M	29.9	None
**De-Identified Patient Code**	**Patient Demographics of Tumor Samples: Patients with Prostate Cancer**			
	**Age at Surgery**	**Sex**	**BMI**	**History of Other Cancers/Pre-Treatment Status at Surgery**
**CTC-PC-M11**	69	M	44.3	None
**CTC-PC-M22**	79	M	31	None
**De-Identified Patient Code**	**Patient Demographics of Tumor Samples: Patients with Esophageal Cancer**			
	**Age at Surgery**	**Sex**	**BMI**	**History of Other Cancers/Pre-Treatment Status at Surgery**
**CTC-EsoC-G11**	66	M	41.2	None

* Patient with African-American ethnicity.

**Table 4:** Grade-wise distribution of patients’ blood samples used for the standardization and testing of CTCs, along with tumors from each pathology.

Grades of Patients with Different Tumors (Endometrial, Ovary, Lung, Esophageal, Prostate, and Liver)	Total Percentage of Patients’ Blood Used for the Study (%)	Number of Blood Samples Used for CTC Standardization		Number of Blood Samples Used for CTC Testing		Percentage of Patients with Positive CTC (IF and/or ICC) (%)
**G1**	47%	5		38		50%
**G2**	18%	4		12		58%
**G3**	20%	2		16		69%
Tumors from Each Organ Type						
**Tumors from Each Pathology**	**Endometrial**	**Ovary**	**Lung**	**Esophageal**	**Prostate**	**Liver**
**G1**	37	5	1	0	0	NA
**G2**	12	0	3	1	0	
**G3**	16	1	0	0	1	

## Data Availability

We have not used any publicly available data. All data presented in the MS is obtained from the patient samples following informed consent from the patient with proper IRB approval.
